# MRI-derived global small vessel disease burden serves as a marker of hippocampal sclerosis and clinical stage across the probable Alzheimer’s disease continuum

**DOI:** 10.3389/fnagi.2025.1692747

**Published:** 2025-11-19

**Authors:** Ya-Che Chen, Ting-Bin Chen, Hsin-Chieh Chen

**Affiliations:** 1 Department of Radiology, Tri-Service General Hospital, National Defense Medical University, Taipei, Taiwan; 2 Department of Neurology, Kuang Tien General Hospital, Taichung, Taiwan; 3 Department of Medical Equipment Development and Application, Hungkuang University, Taichung, Taiwan

**Keywords:** biomarker, hypertensive arteriopathy, amyloid angiopathy, lacune, microbleed, white matter hyperintensity

## Abstract

**Introduction:**

Cerebral small vessel disease (SVD) contributes to cognitive decline and hippocampal sclerosis (HS), yet its role across the Alzheimer’s disease (AD) continuum remains incompletely understood. We aimed to determine whether composite MRI-based SVD scores serve as markers of cognitive impairment and HS in cognitively unimpaired (CU) individuals, patients with mild cognitive impairment (MCI), and those with probable AD dementia.

**Methods:**

We retrospectively analyzed 200 participants (24 CUs, 34 MCI, 142 AD) from the dementia registry at Kuang Tien General Hospital (January 2024–June 2025). SVD burden was quantified using three composite imaging scores: global SVD, cerebral amyloid angiopathy (CAA)-SVD, and hypertensive arteriopathy (HA)-SVD. Associations with cognitive performance, clinical staging, and HS were examined using multivariable regression models.

**Results:**

Global SVD and CAA-SVD scores correlated with Cognitive Abilities Screening Instrument (CASI) total and domain subscores, Clinical Dementia Rating (CDR) global score, and CDR sum of boxes (CDR-SB). Notably, only the global SVD score remained independently associated with both CDR-SB and HS after adjustment for relevant confounders.

**Discussion:**

MRI-derived global SVD burden, reflecting the combined effects of CAA and HA, is strongly associated with cognition, clinical staging, and HS across the probable AD continuum, supporting the global SVD score as a clinically useful biomarker of vascular contributions. Since MCI/AD diagnoses were based on clinical criteria without confirmation using cerebrospinal fluid or positive positron emission tomography biomarkers, potential misclassification may exist; findings should be interpreted with caution.

## Introduction

Alzheimer’s disease (AD) represents a clinically and biologically heterogeneous disorder characterized by frequent co-occurrence of multiple neurodegenerative pathologies (Maldonado-Díaz et al., 2024). Autopsy studies consistently demonstrate that pure AD neuropathologic change is uncommon, with mixed pathologies predominating across the disease spectrum (Thomas et al., 2020). These co-pathologies encompass additional neurodegenerative lesions, including limbic TAR DNA-binding protein-43 (TDP-43) deposition, hippocampal sclerosis (HS), and Lewy body-related pathology, alongside vascular injury (Robinson et al., 2018). The cumulative burden of these multiple pathological processes likely underlies the remarkable variability in clinical phenotype, rate of progression, and treatment response observed along the AD continuum from normal cognition through mild cognitive impairment (MCI) to overt AD dementia.

Among the constellation of AD co-pathologies, cerebral small vessel disease (SVD) is a central—and critically, potentially modifiable—determinant of disease heterogeneity (Toledo et al., 2013; Fisher et al., 2022). SVD and AD share overlapping risk factor profiles, including hypertension, diabetes mellitus, smoking, hyperlipidemia, and apolipoprotein E ε4, while SVD is independently linked to stroke, cognitive decline, gait disturbance, and late-life dementia (Gorelick et al., 2011; Kim et al., 2020). Two partially overlapping but mechanistically distinct processes dominate in aging and AD brains: cerebral amyloid angiopathy (CAA), characterized by β-amyloid deposition in leptomeningeal and cortical vessels with a posterior predominance; and hypertensive arteriopathy (HA), marked by arteriolosclerosis/lipohyalinosis of deep perforators that supply the basal ganglia, thalamus, and deep white matter (Inoue et al., 2023; Hainsworth et al., 2024). These distinct vascular substrates carry differential clinical implications and manifest as recognizable magnetic resonance imaging (MRI) signatures—lobar microbleeds, cortical superficial siderosis, and centrum semiovale enlarged perivascular spaces (EPVS) in CAA; while deep microbleeds, basal ganglia EPVS, lacunes, and confluent deep white matter hyperintensities (WMH) in HA (Duering et al., 2023).

Vascular pathology is the most prevalent coexisting process in AD and likely acts as an additional pathobiological “hit,” lowering the threshold at which AD pathology manifests clinically and accelerating cognitive decline across disease stages (Kapasi and Schneider, 2016; Fisher et al., 2022). Mechanistically, SVD contributes to a cascade of deleterious processes including chronic hypoperfusion, blood–brain barrier (BBB) disruption, glymphatic dysfunction, microinfarction and microhemorrhage, and neuroinflammation, all of which can exacerbate amyloid and tau accumulation, accelerate synaptic loss, and amplify network disconnection (Kalaria and Sepulveda-Falla, 2021; Inoue et al., 2023). Conversely, AD pathology may reciprocally compromise vascular integrity and impair perivascular clearance mechanisms, establishing a bidirectional, self-reinforcing pathological loop (Iturria-Medina et al., 2016; Sweeney et al., 2018; Kim et al., 2020). This dynamic interplay strongly suggests that SVD functions not as a passive bystander but as an active contributor to clinical deterioration throughout the AD continuum.

As singular MRI markers of SVD (i.e., microbleed, lacune, EPVS, and WMH) incompletely characterize the full scope of cerebral microvascular injury, composite SVD scores have been developed and validated in clinical and neuropathological cohorts to provide a more comprehensive assessment (Staals et al., 2014; Charidimou et al., 2016; Lau et al., 2017; Pasi et al., 2021). These composite measures integrate established MRI features of SVD and provide semi-quantitative indices that capture global SVD burden (a whole-brain cumulative microangiopathy), CAA-related SVD burden, and HA-related SVD burden (Charidimou et al., 2016; Lau et al., 2017; Pasi et al., 2021). Higher SVD composites consistently associate with worse global cognition, domain-specific deficits (processing speed, executive function, and memory), functional decline, and gait/balance impairment, underscoring their utility as scalable neuroimaging proxies of cerebral microvascular damage (Charidimou et al., 2016; Lin et al., 2021; Pasi et al., 2021).

Hippocampal sclerosis (HS)—characterized by selective neuronal loss, astrogliosis, microglial activation, and granulovacuolar degeneration in the CA1 region and subiculum—has been increasingly recognized as a pivotal and independent contributor to late-life memory impairment, often coexisting with AD pathology (Nelson et al., 2011; Pao et al., 2011). A body of neuropathological evidence indicates that HS in late-life is a convergent endpoint of three major pathobiological streams: (1) proteinopathies (TDP-43, tau, β-amyloid) disrupt proteostasis and synaptic integrity; (2) cerebrovascular injury mediated through both CAA and HA imposes metabolic stress via hypoperfusion, BBB breakdown, and microinfarction; and (3) intrinsic cellular–structural degeneration in CA1/subiculum constitutes the core histopathological signature of HS (Meneses et al., 2021; Sordo et al., 2023; Wisse et al., 2025). Despite its clinical impact, the relationship between SVD imaging markers and HS occurrence across the AD continuum remains critically underexplored.

We sought to determine whether composite MRI-derived measures of SVD burden—global, CAA-related, and HA-related scores—are associated with cognition, clinical staging, and HS across the probable AD continuum. Using a retrospective cohort of cognitively unimpaired controls (CUs), patients with mild cognitive impairment (MCI), and patients with probable AD dementia, we examined the potential of these indices as translational imaging biomarkers of vascular contributions to AD and HS.

## Methods

### Subjects

Participants included patients with MCI, patients with probable AD, and CUs, retrospectively recruited between January 2024 and June 2025 from the dementia care database at Kuang Tien General Hospital. The study protocol was approved by the Institutional Review Board and Ethics Committee of Kuang Tien General Hospital. All procedures were conducted in accordance with relevant guidelines and regulations.

Inclusion criteria were age ≥55 years and completion of clinical assessment, neuropsychological testing, and brain MRI examination. For each participant, a trained, licensed nurse case manager collected routine demographic and clinical data, including a brief medical history, age, sex, body mass index, documented comorbidities (cardiovascular and metabolic diseases), and cigarette and alcohol use. Vascular risk factors, specifically hypertension, diabetes mellitus, hyperlipidemia, peripheral or cardiac vasculopathy, and atrial fibrillation, were recorded to characterize vascular comorbidity burden. Neuropsychological evaluation included the Cognitive Abilities Screening Instrument (CASI) (Teng et al., 1994) and the Clinical Dementia Rating (CDR) scale (Hughes et al., 1982).

### Clinical diagnosis

Medical records and registry data were reviewed in detail. Cognitive status was assessed through comprehensive evaluations, including clinical interviews, neurological examinations, neuropsychological testing, functional assessments, brain MRI, and blood analyses. Diagnoses of MCI and probable AD were made according to the core clinical criteria established by the 2011 National Institute on Aging–Alzheimer’s Association workgroups (Albert et al., 2011; McKhann et al., 2011). Cognitively unimpaired controls were recruited from individuals attending the neurology clinic who demonstrated normal cognition on the same comprehensive assessments. Participants with cognitive impairment due to non-AD etiologies, such as brain tumor, stroke, Parkinson’s disease, thyroid dysfunction, renal insufficiency, unstable diabetes mellitus, vitamin B12 deficiency, neurosyphilis, major psychiatric illness, or any other systemic diseases that might affect cognitive function, were excluded.

### Brain MRI

The 1.5-T MRI protocol (MAGNETOM Sola, Siemens Healthcare, Germany) included the following standardized sequences: axial spin echo (SE) T1-weighted imaging (repetition time (TR)/echo time (TE) = 550/8.9 ms, field of view (FOV) = 230 × 230 mm^2^, matrix = 320 × 256, slice thickness = 6 mm); axial turbo spin echo (TSE) T2-weighted imaging (T2WI) (TR/TE = 3,500/103 ms, FOV = 230 × 208 mm^2^, matrix = 512 × 384, slice thickness = 6 mm); oblique coronal T2-weighted TSE imaging perpendicular to the hippocampal axis (TR/TE = 4,000/83 ms, FOV = 180 × 180 mm^2^, matrix = 384 × 307, slice thickness = 3 mm); axial fluid-attenuated inversion recovery (FLAIR) imaging (TR/TE/inversion time = 9,000/86/2,500 ms, FOV = 230 × 201 mm^2^, matrix = 320 × 240, slice thickness = 6 mm); axial diffusion-weighted imaging (TR/TE = 6,300/89 ms, FOV = 230 × 230 mm^2^, matrix = 192 × 192, slice thickness = 6 mm); axial susceptibility-weighted imaging (SWI) (TR/TE = 49/40 ms, FOV = 230 × 186 mm^2^, flip angle = 15°, matrix = 288 × 230, slice thickness = 2 mm); and three-dimensional time-of-flight magnetic resonance angiography (TR/TE = 24/7 ms, FOV = 180 × 180 mm^2^, matrix = 256 × 218, flip angle = 25°, slice thickness = 0.5 mm, 4 slabs with 52 slices per slab).

### Visual rating scales

The MRI data were rated by an experienced neuroradiologist (Ya-Che Chen) who was blinded to participant diagnoses. The details of the imaging scoring systems have been described in our previous studies (Lin et al., 2021; Chen et al., 2022). HS was defined as hippocampal atrophy with hyperintense signal on long-repetition-time sequences (T2/FLAIR) confined to the hippocampus, consistent with prior standards (Jackson et al., 1993; Bronen, 1998). Regional brain atrophy was measured with visual rating scales based on the T1-weighted images. Medial temporal atrophy (MTA), posterior atrophy (PA), and frontal lobe atrophy were assessed with the MTA scale (0–4) (Scheltens et al., 1992), Koedam’s scale (0–3) (Koedam et al., 2011), and global cortical atrophy scale–frontal subscale (GCA-F) (0–3) (Ferreira et al., 2016), respectively.

SVD imaging markers were assessed according to the STRIVE consensus (Wardlaw et al., 2013). Cerebral microbleeds (CMBs), recognized as ≤10 mm diameter circular hypointense lesions on SWI, were counted in lobar, deep, and infratentorial regions, recorded based on the Microbleed Anatomical Rating Scale (Gregoire et al., 2009). The total CMB burden was calculated as the sum across all regions. WMHs defined by ill-defined hyperintensities ≥5 mm across on FLAIR images and graded according to the age-related White Matter Change (ARWMC) scale (0–3) were summed to represent overall WMH burden (Wahlund et al., 2001). Periventricular and deep WMHs were also graded separately using the modified Fazekas scale (0–3) (Fazekas et al., 1987). Enlarged perivascular spaces (PVSEs) were identified as T2-weighted hyperintense lesions >3 mm with cerebrospinal fluid–like signal intensity (Doubal et al., 2010). They were counted in the basal ganglia (BG) and centrum semiovale (CSO) and rated as follows: 0, none; 1, 1–10; 2, 11–20; 3, 21–40; 4, >40. Hippocampal PVSE were counted bilaterally, and the summed values represented the total hippocampal PVSE count (Pan et al., 2025). Lacunes were counted across the whole brain on FLAIR sequence images. Curvilinear hypointensities following the gyral surface on SWI were recorded as cortical superficial sideroses (CSSs).

### SVD composite scoring

To quantify CAA-related, HA-related, and whole-brain SVD burden, we employed three validated composite scores (Staals et al., 2014; Charidimou et al., 2016; Lau et al., 2017; Pasi et al., 2021). The CAA-SVD score captures the canonical MRI signature of CAA and has been validated against neuropathological CAA severity and clinical phenotype (Charidimou et al., 2016). The HA-SVD score, derived from the SVD framework and demonstrating convergent validity with vascular risk profiles and lacunar stroke subtypes, was adapted to emphasize hypertensive arteriopathy (Staals et al., 2014). The global SVD score integrates both hemorrhagic and ischemic substrates to quantify cumulative microangiopathy burden and has demonstrated external prognostic validity for cognitive decline following intracerebral hemorrhage and for recurrent stroke in large cohorts (Lau et al., 2017; Pasi et al., 2021).

Briefly, the global SVD score (0–6) assigned 1 point each for 1–4 lobar CMBs, ≥1 lacune, ≥20 BG PVSEs, and moderate WMH (total periventricular + deep WMH grade 3–4), and 2 points each for ≥5 CMBs or severe WMH (grade 5–6). CAA-SVD score (0–6) assigned 1 point each for 2–4 lobar CMBs, ≥20 CSO PVSEs, ≥2 deep WMH or 3 periventricular WMH, or focal CSSs, and 2 points each for ≥5 lobar CMBs or disseminated CSSs. HA-SVD score (0–4) assigned 1 point each for ≥1 deep CMB, ≥1 lacune, ≥10 BG PVSEs, and ≥2 deep WMH or 3 periventricular WMH.

### Statistical analysis

Baseline clinical characteristics of subjects with and without HS were compared using independent sample t-tests for continuous variables (e.g., age, scales, and scores), the Mann–Whitney U test for skewed continuous variables (i.e., total cerebral microbleeds), and Chi-square tests for categorical variables (e.g., sex and clinical stage). The relationship between the three composite SVD scores (i.e., global, CAA, and HA) and the total and domain subscores of the CASI, CDR, and CDR sum of boxes (CDR-SB) was assessed using Spearman’s rank correlation. The association of composite SVD scores with CDR-SB was assessed using linear regression models. Furthermore, the association of composite SVD scores with the presence of HS was evaluated using logistic regression models. A two-step adjustment approach was applied in all regression models. The first step adjusted for age, sex, and years of education. In the second step, linear regression models were further adjusted for the number of vascular risk factors, whereas logistic regression models additionally included both the number of vascular risk factors and the CDR score. These covariates were selected based on their established clinical relevance as potential confounders in the associations between SVD burden, cognitive outcomes, and hippocampal pathology. Multicollinearity was assessed using variance inflation factors (VIFs); VIF values <10 were considered to indicate acceptable variable independence. Analyses were performed in SPSS version 26.0 for Windows (SPSS Inc., Chicago, IL). All statistical tests were two-tailed with a significance threshold of *p* < 0.05.

## Results

### Clinical and imaging data of the entire cohort

A total of 200 subjects were enrolled in the study and divided into HS− and HS+ groups. The clinical characteristics of the study cohort are summarized in Table 1. There were no significant between-group differences in age, sex, years of education, clinical stage, or overall vascular comorbidity. Compared to the HS− group, the HS+ group had higher levels in GCA-F (1.71 ± 0.61 vs. 1.23 ± 0.78, *p* < 0.001), PA (1.65 ± 0.65 vs. 1.21 ± 0.77, *p* < 0.001), average MTA (2.45 ± 0.70 vs. 0.51 ± 0.53, *p* < 0.001), periventricular WMH (1.81 ± 0.69 vs. 1.47 ± 0.77, *p* = 0.004), hippocampal enlarged PVS (8.3 ± 4.8 vs. 2.3 ± 2.1, *p* = 0.007), CDR (0.85 ± 0.52 vs. 0.62 ± 0.47, *p* = 0.002), and CDR-SB (4.7 ± 3.3 vs. 3.0 ± 2.6, *p* < 0.001), but lower performance on CASI (58.6 ± 23.0 vs. 68.9 ± 20.1, *p* = 0.002). Regarding cognition, the HS + group had worse ability in long-term memory, short-term memory, mental manipulation, orientation, abstraction, and language than the HS– group (Supplementary Table S1).

**Table 1:** Clinical characteristics of the study cohort with or without hippocampal sclerosis.

Variable	Total (*n* = 200)	HS (+) (*n* = 63)	HS (−) (*n* = 137)	*P* value
Demographics				
Sex, male	80 (40.0)	31 (49.2)	49 (35.8)	0.072
Age, year	75.0 ± 8.3	76.4 ± 9.0	74.3 ± 7.8	0.092
Education level, year	7.3 ± 4.8	6.9 ± 4.7	7.5 ± 4.8	0.375
Hypertension	81 (40.5)	17 (27.0)	64 (46.7)	0.008
Clinical stage				0.153
Cognitively unimpaired	24 (12.0)	4 (6.3)	20 (14.6)	
Mild cognitive impairment	34 (17.0)	9 (14.3)	25 (18.2)	
Alzheimer’s disease dementia	142 (71.0)	50 (79.4)	92 (67.2)	
Diabetes mellitus	54 (27.0)	15 (23.8)	39 (28.5)	0.491
Hyperlipidemia	55 (27.5)	11 (17.5)	44 (32.1)	0.031
Peripheral or cardiac vasculopathy*	19 (9.5)	5 (7.9)	14 (10.2)	0.609
Atrial fibrillation	9 (4.5)	4 (6.3)	5 (3.6)	0.392
Number of vascular risk factors				0.077
0	100 (50.0)	40 (63.5)	60 (43.8)	
1	32 (16.0)	7 (11.1)	25 (18.2)	
2	27 (13.5)	7 (11.1)	20 (14.6)	
≥3	41 (20.5)	9 (14.3)	32 (23.4)	
Imaging data				
Global cortical atrophy-frontal subscale	1.39 ± 0.76	1.71 ± 0.61	1.23 ± 0.78	<0.001
Posterior atrophy	1.35 ± 0.76	1.65 ± 0.65	1.21 ± 0.77	<0.001
Average medial temporal atrophy	1.12 ± 1.08	2.45 ± 0.70	0.51 ± 0.53	<0.001
Total cerebral microbleed	0.0 [0.0, 1.0]	0.0 [0.0, 1.0]	0.0 [0.0, 1.0]	0.521
Lacune	1.16 ± 2.08	1.43 ± 2.76	1.04 ± 1.67	0.216
Periventricular WMH	1.58 ± 0.76	1.81 ± 0.69	1.47 ± 0.77	0.004
Deep WMH	1.33 ± 0.89	1.40 ± 0.96	1.30 ± 0.85	0.471
Total WMH burden	5.5 ± 3.1	5.9 ± 3.4	5.4 ± 3.0	0.291
PVSE in centrum semiovale	2.87 ± 0.97	2.89 ± 0.97	2.86 ± 0.97	0.852
PVSE in basal ganglia	2.6 ± 0.9	2.7 ± 0.8	2.5 ± 0.9	0.119
Hippocampal PVSE	4.2 ± 4.2	8.3 ± 4.8	2.3 ± 2.1	<0.001
Global SVD score	2.1 ± 1.5	2.5 ± 1.3	1.9 ± 1.5	0.007
Cerebral amyloid angiopathy-SVD score	1.26 ± 0.97	1.37 ± 0.99	1.20 ± 0.96	0.276
Hypertensive arteriopathy-SVD score	2.6 ± 1.7	2.9 ± 2.0	2.5 ± 1.6	0.129
Cognitive data				
Cognitive abilities screening instrument	65.6 ± 21.5	58.6 ± 23.0	68.9 ± 20.1	0.002
Clinical dementia rating	0.69 ± 0.50	0.85 ± 0.52	0.62 ± 0.47	0.002
Clinical dementia rating sum of boxes	3.6 ± 2.9	4.7 ± 3.3	3.0 ± 2.6	<0.001

HS, hippocampal sclerosis; WMH, white matter hyperintensity; PVSE, enlarged perivascular space; SVD, small vessel disease.

*Including carotid stenosis, coronary artery disease, myocardial infarction and peripheral arterial occlusive disease.

Data were presented as frequency (percentage), mean ± standard deviation or median [25th percentile, 75th percentiles].

Association of composite SVD scores with global cognition and cognitive domains

As shown in Table 2, the global SVD score was significantly correlated with the total CASI score and all nine domain subscores, as well as with CDR and CDR-SB scores. The CAA-SVD score showed significant correlations with the total CASI score and seven domain subscores—excluding mental manipulation and visual construction. In contrast, the HA-SVD score was only correlated with CDR-SB. Overall, both global SVD and CAA-SVD scores were negatively associated with cognitive performance (CASI) and positively associated with clinical dementia severity (CDR and CDR-SB).

**Table 2:** Correlation of imaging variables with cognitive performance.

	Composite SVD score					
	Global SVD		CAA-SVD		HA-SVD	
Cognitive parameters	*ρ*	*P* value	*ρ*	*P* value	*ρ*	*P* value
CDR	0.27	<0.001	0.19	0.008	−0.10	0.151
CDR-SB	0.29	<0.001	0.20	0.005	−0.15	0.036
CASI	−0.28	<0.001	−0.19	0.007	0.04	0.532
Long-term memory	−0.24	0.001	−0.18	0.011	−0.03	0.700
Short-term memory	−0.19	0.008	−0.14	0.047	0.05	0.462
Attention	−0.25	<0.001	−0.14	0.045	0.02	0.795
Mental manipulation	−0.16	0.026	−0.11	0.123	0.03	0.678
Orientation	−0.22	0.002	−0.17	0.014	0.11	0.135
Abstraction	−0.21	0.003	−0.18	0.010	0.04	0.535
Language	−0.31	<0.001	−0.24	0.001	−0.09	0.208
Visual construction	−0.21	0.003	−0.12	0.090	0.00	0.961
Verbal fluency	−0.28	<0.001	−0.22	0.002	−0.01	0.881

SVD, small vessel disease; CAA, cerebral amyloid angiopathy; HA, hypertensive arteriopathy; CDR, clinical dementia rating; CDR-SB, clinical dementia rating sum of boxes; CASI, cognitive abilities screening instrument.

### Association of composite SVD scores with CDR-SB and hippocampal sclerosis

**Figure 1:**
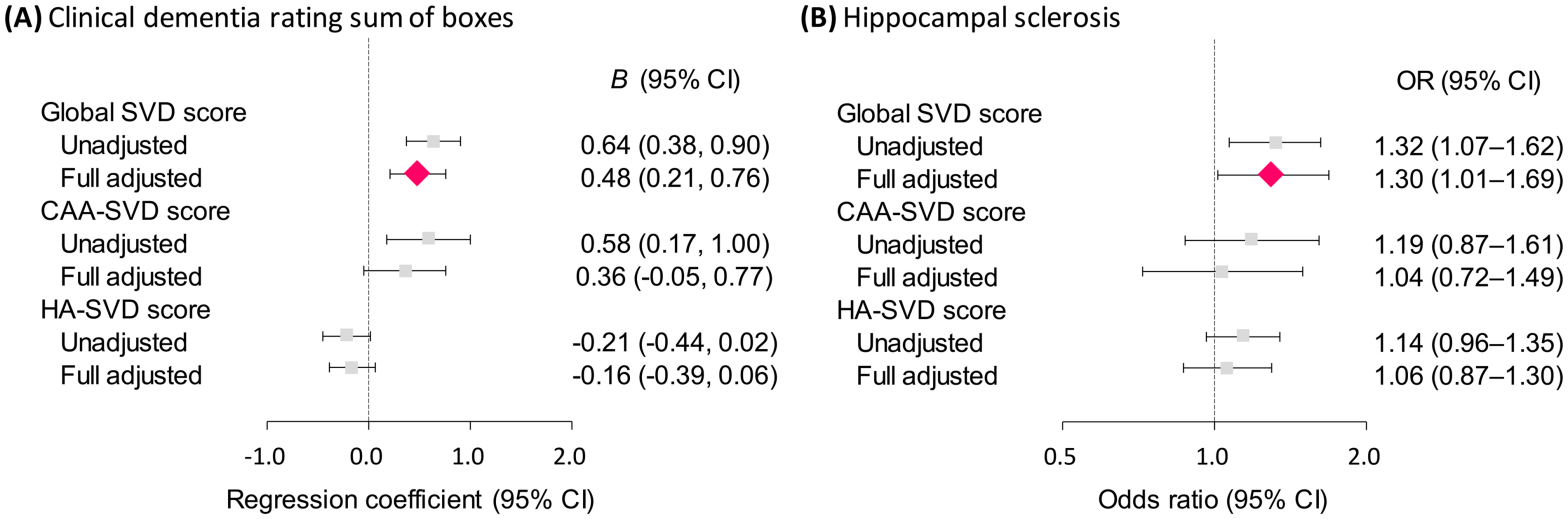
Forest plot showing the associations of composite SVD scores with CDR-SB **(A)** and hippocampal sclerosis **(B)**. SVD, small vessel disease; CDR-SB, clinical dementia rating sum of boxes; *B*, regression coefficient; CI, confidence interval; OR, odds ratio.

Among the three composite SVD scores, only the global SVD score remained significantly and positively associated with CDR-SB, even after two-step statistical adjustment for demographic variables and vascular risk burden (regression coefficient = 0.48, 95% confidence interval [CI]: 0.21 to 0.76; as shown in Table 3 and Figure 1A). Similarly, the global SVD was the only score that retained a significant association with hippocampal sclerosis in the fully adjusted logistic regression model (odds ratio = 1.30, 95% CI: 1.01–1.69; as presented in Table 4 and Figure 1B), underscoring its robust and independent clinical relevance after accounting for potential confounders. All independent variables had VIF values well below the threshold of 10 (all <2.0), indicating no substantial multicollinearity (data not shown).

**Table 3:** Associations of composite small vessel disease scores with CDR-SB.

Variable/model	Regression coefficient (95% CI)	*P* value
Global small vessel disease score		
Unadjusted	0.64 (0.38, 0.90)	<0.001
Model 1	0.49 (0.21, 0.77)	0.001
Model 2	0.48 (0.21, 0.76)	0.001
CAA-small vessel disease score		
Unadjusted	0.58 (0.17, 1.00)	0.006
Model 1	0.34 (−0.08, 0.76)	0.109
Model 2	0.36 (−0.05, 0.77)	0.088
HA-small vessel disease score		
Unadjusted	−0.21 (−0.44, 0.02)	0.078
Model 1	−0.24 (−0.46, −0.02)	0.032
Model 2	−0.16 (−0.39, 0.06)	0.159

CDR-SB, clinical dementia rating sum of boxes; CI, confidence interval; CAA, cerebral amyloid angiopathy; HA, hypertensive arteriopathy.

Model 1: adjusted for age, sex and education level.

Model 2: further adjusted for number of vascular risk factors.

**Table 4:** Associations of composite small vessel disease scores with the likelihood of hippocampal sclerosis.

Variable/model	Odds ratio (95% CI)	*P* value
Global small vessel disease score		
Unadjusted	1.32 (1.07–1.62)	0.008
Model 1	1.28 (1.02–1.62)	0.034
Model 2	1.30 (1.01–1.69)	0.045
CAA-small vessel disease score		
Unadjusted	1.19 (0.87–1.61)	0.275
Model 1	1.07 (0.77–1.49)	0.699
Model 2	1.04 (0.72–1.49)	0.845
HA-small vessel disease score		
Unadjusted	1.14 (0.96–1.35)	0.131
Model 1	1.11 (0.93–1.33)	0.253
Model 2	1.06 (0.87–1.30)	0.549

CI, confidence interval.

Model 1: adjusted for age, sex and education level.

Model 2: further adjusted for number of vascular risk factors and clinical dementia rating.

## Discussion

In this retrospective cohort study spanning the probable AD continuum, we examined the relationships between MRI-derived SVD burden indices, cognitive function, and HS. Higher global SVD and CAA-SVD scores were consistently associated with poorer cognitive performance. Among the three composite indices, the global SVD score emerged as the most robust indicator of both clinical stage and HS. These findings suggest that the global SVD score captures the cumulative vascular impact of amyloid- and hypertension-related microangiopathies on cognitive decline and HS, underscoring its promise as an imaging biomarker of vascular contributions to AD. To our knowledge, this is the first study to demonstrate subtype-specific SVD imaging burden as a determinant of cognition, clinical staging, and HS across the probable AD continuum.

Accumulating evidence indicates that both singular SVD imaging markers and composite imaging measures are strongly related to cognition in the population with and without cognitive impairment (Jansma et al., 2024; Charisis et al., 2025). Both global SVD and CAA-SVD composite scores were correlated with worse global and nine domain-specific cognitive functions in the present study, in line with previous studies on MCI, AD, and vascular dementia, showing the associations of global and domain-specific cognitive profiles with the two composite scores (Matsuda et al., 2021; Lin et al., 2021; Chen et al., 2022). Moreover, among the three composite markers, the global SVD score showed the most robust association with cognitive performance, reinforcing the concept that the aggregate burden of both CAA and non-amyloid HA is a key driver of cognitive deterioration across the AD spectrum. Mechanistically, the combined influence of chronic hypoperfusion, microhemorrhages, BBB disruption, and impaired perivascular clearance—features captured by the global score—likely accelerates amyloid and tau deposition, synaptic loss, and network disconnection, thereby lowering the threshold for clinical dementia (Kim et al., 2022). The total burden of coexisting microvascular pathological abnormalities appears to be a key determinant of cognitive impairment and may provide important insights into the interplay between vascular pathology and cognitive decline over the AD continuum.

SVD pathologies are highly prevalent in the aging brain and contribute significantly to the development and progression of AD (Sun et al., 2023). Pathology studies suggest that CAA is nearly ubiquitous in AD brains (severe CAA in 30–60%), while HA is present in roughly half of AD cases (moderate–severe HA in 20–40%), particularly in those with coexisting vascular risk factors (Arvanitakis et al., 2016; Kalaria and Sepulveda-Falla, 2021). The CAA-SVD score aligns more closely with amyloid-related pathology and lobar microvascular injury, while the HA-SVD score reflects hypertensive arteriopathy, predominantly affecting deep brain structures. In our study, unlike the HA-SVD score, the CAA-SVD score was associated with cognition, suggesting differential roles of CAA and HA pathologies in contributing to cognitive decline across the probable AD continuum. Based on our current and prior imaging findings, we infer that (1) CAA burden progressively increases and correlates with cognition across the probable AD continuum—from normal cognition to MCI, and ultimately to mild dementia, (2) HA burden is mainly responsible for cognitive progression in dementia stage, and (3) both CAA and HA exert synergistic effects on cognitive decline and atrophic patterns (Chen et al., 2022). These findings support the notion that distinct types of cerebral microangiopathies may differentially contribute to cognitive and neurodegenerative processes, highlighting the heterogeneity and complexity of vascular contributions in AD.

Across imaging modalities, measures of either neurodegenerative changes (atrophy, glucose hypometabolism, or tau accumulation) or vascular injury (WMH or SVD composite measures) were reported to correlate with higher CDR-SB or predict steeper AD progression (Kamal et al., 2023; Yang et al., 2023; Du et al., 2025). Of the three composite measures, only the global SVD score emerged as an independent indicator of clinical dementia stage. This finding underscores the importance of cumulative microvascular burden, including lacunes, microbleeds, white-matter hyperintensities, and enlarged perivascular spaces, as a critical driver of cognitive and functional impairment in AD. By integrating multiple vascular substrates into a single metric, the global score likely offers a more comprehensive “vascular hit” on neural networks, providing an informative biomarker for staging, prognosis, and future therapeutic targeting of vascular contributions to the AD continuum.

HS in late life appears to arise from converging protein, vascular, and cellular stressors, rather than a single etiologic pathway. Synergistic interplay among these insults lowers the threshold for HS, transforming it from a secondary finding into a pivotal contributor to cognitive decline in AD. Of the three composite scores, only the global SVD score was an independent marker of the presence of HS. Recent large-scale neuropathological analyses indicated that limbic-predominant age-related TDP-43 encephalopathy neuropathologic change (LATE-NC) is the primary driver of hippocampal sclerosis in advanced age (Woodworth et al., 2025). Conversely, the apparent links between HS and either AD neuropathologic change or limbic Lewy bodies were markedly attenuated and became non-significant after adjusting for LATE-NC, consistent with mediation through LATE pathology (Woodworth et al., 2025). In contrast, global cerebrovascular neuropathologic changes remained independently associated with HS even after accounting for LATE-NC, underscoring a parallel vascular pathway to HS (Woodworth et al., 2025). At the extreme, the predicted probability of HS approached ~95% in individuals with LATE-NC stage 3 and severe global cerebrovascular pathology, highlighting the convergent impact of proteinopathy and SVD on CA1/subicular vulnerability (Woodworth et al., 2025). This perspective also motivates vascular-directed prevention and adjunctive therapeutic strategies (e.g., hypertension control and cerebrovascular risk reduction) to complement proteinopathy-targeted approaches, with the goal of mitigating HS-related cognitive decline in AD.

Although the per-point association between the global SVD score and HS appears modest (OR = 1.30; 95% CI: 1.01–1.69), its clinical impact is multiplicative across the full 0–6 scale: increases of 2, 3, 4, and 6 points correspond to approximately 1.69-, 2.20-, 2.86-, and 4.83-fold higher odds of HS, respectively. Given the high prevalence of SVD burden in aging populations, even incremental per-point effects translate into clinically meaningful risk gradients at both individual and population levels. From a clinical perspective, HS is a common and prognostically adverse lesion in older adults. The independent association between global SVD burden and HS supports a mechanistic link between chronic microvascular injury and hippocampal neurodegeneration, potentially mediated through hypoperfusion, BBB dysfunction, and impaired metabolic clearance. The global SVD score—readily derivable from routine MRI sequences—therefore offers a practical tool with multiple clinical applications: (1) risk stratification to identify patients with moderate-to-severe SVD burden who may warrant closer cognitive monitoring and more aggressive vascular risk factor management; (2) informing prognosis discussions with patients and families; and (3) enriching enrollment in clinical trials targeting mixed-pathology dementia. As precision medicine approaches evolve, incorporating this scalable vascular biomarker alongside proteinopathy-directed therapies may help tailor prevention and treatment strategies for individuals across the AD continuum.

Our data—showing an independent association between the global SVD score and HS—support a convergent model in which microvascular injury lowers the threshold for CA1/subicular neurodegeneration through several complementary pathways. First, hemodynamic insufficiency in SVD produces chronic hypoperfusion with BBB disruption, metabolic stress, and impaired waste clearance in the hippocampus; these processes preferentially involve medial temporal regions that exhibit selective vulnerability to ischemic injury (Wong et al., 2019; Johnson, 2023). Second, SVD-related perivascular clearance failure, endothelial dysfunction, and neuroinflammation further amplify proteostatic stress, rendering the CA1/subicular fields particularly susceptible to the neuronal loss and gliosis that characterize HS (Inoue et al., 2023). Third, neuropathological studies demonstrate that arteriolosclerosis—a core SVD lesion—associates quantitatively with both HS and LATE-NC, supporting a direct vascular contribution beyond purely proteinopathic mechanisms (Sordo et al., 2023).

Regarding the interplay between SVD and LATE-NC, LATE-NC is strongly associated with arteriolosclerosis and independently linked to dementia, suggesting biological crosstalk between vascular injury and TDP-43 pathways (Harrison et al., 2021). HS and TDP-43 pathology frequently co-occur, and their combined presence relates to disproportionately worse cognitive outcomes, indicating synergistic rather than redundant effects (Nag et al., 2015). These observations support a “two-hit” interaction: SVD initiates and sustains hippocampal hypoperfusion, BBB dysfunction, and inflammatory signaling, facilitating the emergence or spread of limbic TDP-43 pathology; in turn, LATE-NC magnifies neuronal vulnerability, culminating in HS. Our imaging findings extend this neuropathological framework to the *in vivo* setting, implicating cumulative SVD burden as a clinically accessible marker of the vascular component of HS pathogenesis and highlighting opportunities for vascular risk factor management to mitigate HS-related cognitive decline even with coexisting LATE-NC.

This study has several limitations. First, the cohort comprised consecutive patients with MCI and probable AD diagnosed on clinical grounds without cerebrospinal fluid or amyloid/tau positron emission tomography confirmation. The absence of biomarker-based verification introduces diagnostic heterogeneity and the possibility of misclassification. Inadvertent inclusion of non-AD or mixed dementia syndromes—particularly those with substantial vascular contributions—could overestimate the observed relationships between SVD burden and cognition, clinical stage, or HS. Conversely, inclusion of amyloid-negative subjects could attenuate true associations, biasing results toward the null and yielding conservative estimates. Nevertheless, our primary objective was to evaluate the clinical relevance of MRI-derived SVD indices across a real-world, clinically probable AD continuum, where biomarker data are often unavailable. Within this context, the consistent associations of the global SVD score with cognition, clinical staging, and HS remain informative. Future work should replicate these findings in biomarker-defined AT(N) cohorts and/or incorporate fluid biomarkers to refine disease attribution and quantify the independent vascular contribution with greater specificity. Second, the AD group (*n* = 142) was substantially larger than CU (*n* = 24) and MCI (*n* = 34). This spectrum imbalance may constrain generalizability—especially to earlier disease stages—could reduce precision of estimates in the CU and MCI groups, and potentially amplify stage-driven associations between SVD burden and clinical outcomes. Third, as multiple statistical tests were performed, the possibility of Type I error inflation cannot be excluded. Findings with marginal statistical significance should therefore be interpreted with caution. Future studies with larger sample sizes and appropriate adjustments for multiple testing are needed to validate these associations. Fourth, the retrospective, cross-sectional design precludes causal inference. Longitudinal, biomarker-confirmed studies are required to determine the temporal progression and refine the mechanistic understanding of these associations.

## Conclusion

Across the probable AD continuum, MRI-derived global SVD burden was strongly associated with cognitive performance, clinical staging, and HS. Our findings indicate that the coexistence of HA and CAA accelerates disease progression, underscoring the global SVD score as a practical, integrative imaging biomarker for quantifying vascular contributions to AD and HS and for informing future vascular-targeted prevention strategies.

## Data Availability

The raw data supporting the conclusions of this article will be made available by the authors, without undue reservation.
