# Supplementary material for: MRI-derived global small vessel disease burden serves as a marker of hippocampal sclerosis and clinical stage across the probable Alzheimer’s disease continuum
Source: Front Aging Neurosci. 2025 Nov 19;17:1692747. doi: 10.3389/fnagi.2025.1692747 (PMC12672439; doi:10.3389/fnagi.2025.1692747)
Supplement: Supplementary file 1 [file Table_1.docx]

**Supplemental Table 1.** The cognitive domains of CASI of the cohort with or without hippocampal sclerosis

| Variable | Total  (*n* = 200) | HS (+)  (*n* = 63) | HS (-)  (*n* = 137) | *P* value |
| --- | --- | --- | --- | --- |
| Long-term memory | 8.4 ± 2.6 | 7.6 ± 3.1 | 8.8 ± 2.2 | 0.001 |
| Short-term memory | 5.6 ± 3.5 | 4.9 ± 3.4 | 6.0 ± 3.5 | 0.034 |
| Attention | 5.7 ± 2.0 | 5.4 ± 2.1 | 5.9 ± 1.9 | 0.075 |
| Mental manipulation | 5.5 ± 3.6 | 4.4 ± 3.9 | 6.1 ± 3.3 | 0.002 |
| Orientation | 11.1 ± 5.2 | 9.6 ± 5.4 | 11.8 ± 4.9 | 0.004 |
| Abstraction | 7.7 ± 2.7 | 6.8 ± 2.9 | 8.1 ± 2.6 | 0.002 |
| Language | 8.3 ± 2.1 | 7.7 ± 2.3 | 8.5 ± 1.9 | 0.013 |
| Visual construction | 8.1 ± 2.7 | 7.6 ± 3.0 | 8.2 ± 2.5 | 0.129 |
| Verbal fluency | 5.1 ± 2.9 | 4.7 ± 2.9 | 5.4 ± 2.9 | 0.102 |

Abbreviation: CASI, cognitive abilities screening instrument; HS, hippocampal sclerosis;

Data were presented as mean ± standard deviation.
